# Supplementary material for: Learning to estimate sample-specific transcriptional networks for 7,000 tumors
Source: Proc Natl Acad Sci U S A. 2025 May 23;122(21):e2411930122. doi: 10.1073/pnas.2411930122 (PMC12130817; doi:10.1073/pnas.2411930122)
Supplement: Supplementary file 1 — Appendix 01 (PDF) [file pnas.2411930122.sapp.pdf]

Supporting Information for

Learning to Estimate Sample-specific Transcriptional Networks for 7000 Tumors

C.N. Ellington, B.J. Lengerich, T.B.K. Watkins, J. Yang, A. Adduri, S. Mahbub, H. Xiao, M. Kellis, and E.P. Xing

Corresponding Authors Eric P. Xing and Manolis Kellis.
E-mails: epxing@cs.cmu.edu, manoli@mit.edu

This PDF file includes:

Supporting text
Figs. S1 to S6
Tables S1 to S3
Legends for Dataset S1 to S2
SI References

Other supporting materials for this manuscript include the following:

Datasets S1 to S2

Supporting Information Text

1. Resources and Reproducibility

- Contextualized documentation and installation instructions
  https://contextualized.ml/

- Code for model training, plotting, and evaluation
  https://github.com/cnellington/CancerContextualized

- Preprocessed data and contextualized networks for all 7997 patients with split labels
  https://zenodo.org/records/14885352

- TCGA data download portal
  https://portal.gdc.cancer.gov/

- TCGA reference subtypes
  https://bioconductor.org/packages/3.21/bioc/vignettes/TCGAbiolinks/inst/doc/subtypes.html

2. Implementation

Our entire framework is implemented in PyTorch using the PyTorch Lightning framework within our open-source software Contextualized (1). The context encoder, network archetypes, and contextualized network models are learned simultaneously using end-to-end backpropagation of the network loss (defined in Methods).

Training The context data views (B) were concatenated sample-wise to create a single context feature vector encompassing all views for each patient. Categorical features were one-hot encoded. Healthy samples were given ideal genomic profiles with no SCNAs or SNVs. Other missing values were imputed with the feature mean. The full context vectors were compressed to 200 features using a PCA learned on the training-validation split. We split our dataset into 80% training-validation and 20% testing. We created 30 bootstraps of the training-validation set and finally split into 80% training and 20% validation, resulting in a 64-16-20 split for train-validation-test.

We bootstrapped our models, sampling the train-validation split with replacement to create 30 bootstraps. For each bootstrap, we also initialized the models randomly. We trained the models using a batch-size of 10 and a learning rate of 1e-3. We used early-stopping with a patience of 5 to end training when the minimum validation loss had not improved for 5 epochs. We retain only the model with the minimum validation loss for each bootstrap.

Following this, we use the trained models to infer 30 networks (one from each bootstrap) for each sample in the original non-bootstrapped training-validation set as well as the test set. To obtain the final predicted networks for each sample, we average the network parameters over the bootstraps to get a single bootstrap-averaged network for each sample. The mean-squared errors of the network relative to the sample expression is defined in the Methods (the mean of the linear residuals in the network objectives). We report the mean error of these networks averaged over samples in the test set. Rather than bootstrapping this entire procedure again to report the standard deviation of this result, we upper-bound the uncertainty by reporting the standard deviation of the errors from the individual bootstrap models. This procedure is repeated for all of the baseline methods. All plots can be reproduced using the error files uploaded to Zenodo with the pre-trained networks.

Additionally, we tested both gradient-based optimization and off-the-shelf SKLearn solvers for the baselines where possible, applying early stopping with the gradient-based solvers to control overfitting across all methods. However, we found that the SKLearn solvers were superior when applicable, and reported the best baseline results.

Context Encoder The context encoder is implemented as a multi-layer perceptron with 3 hidden layers, each 100 neurons wide with ReLU activations. Model weights are initialized as `Uniform[-0.01, 0.01]`.

The context encoder is a highly flexible component of our framework and a driving force for future work. It can be used to enforce assumptions about the relationships between contexts and models, between context features, and about the archetype space. For instance, by using a multi-layer perceptron in this study, we naturally handle colinearities in a high-dimensional feature space. Using a neural additive model (2, 3) instead of a multi-layer perceptron would provide context-feature-specific archetype weights for interpretability. Similarly, the context encoder can be implemented as a convolutional neural network for images (4) or a recurrent neural network for timezeries (5). At the context encoder head, we currently use an unconstrained output, but applying a softmax activation would require all of the sample-specific models to lie within a polytope defined by the archetypal networks.

Hardware Limitations 50 metagenes were chosen due to hardware limitations, as the largest number of metagenes we tried which would fit in memory for training and inference on a MacBook Air 2020 with 16G RAM in 32-bit precision. This occupied 11.4G of memory while writing 30 bootstraps * 7997 samples * 50^2 sample-specific network parameters to disk.

3. Data

A. Data sources. The Cancer Genome Atlas[*] (TCGA) is a publicly-available pan-cancer datasource containing genomic, transcriptomic, and clinical profiling of tumors from dozens of landmark studies. We queried TCGA for open access samples with bulk RNA-sequencing and merged this dataset with two follow-up studies on an overlapping set of patients.

Somatic copy number alterations (SCNAs) SCNAs affect a larger fraction of the genome than do any other type of somatic genetic alteration (6) and are a major driver of expression variation in cancer (7). We used copy number profiles derived from TCGA samples using ASCAT (8) from a pan-cancer study of the role of allele-specific SCNAs in cancer (9).

Driver single-nucleotide mutations (SNVs) SNVs can be classified into "driver" mutations thought to provide selective growth advantage and "passenger" mutations thought to have little role in promoting cancer development. We incorporated driver SNVs from the TCGA-derived CHASMplus dataset (10)

B. Context Data Views.

Clinical information This data view incorporates sample tissue-of-origin, race, age at diagnosis, gender, year of birth, and days to collection provided by TCGA.

Biopsy Composition This data view contains the sample's percent tumor cells, percent normal cells, percent tumor nuclei, percent monocyte infiltration, percent lymphocyte infiltration, and percent neutrophil infiltration provided by TCGA. We also incorporate expression-derived estimates of the fraction of a sample consisting of tumor cells from (9).

Copy Number Alterations From ASCAT (8), we gather whole genome doubling events as well as gain and loss events for bp-specific regions of hg19 based on data from (11). We transform these gain and loss events into both arm-level and gene-level events, where arm-level events affect 85% of an entire arm in the same event, while genes-level events affect a single gene. We transform these into number of major and number of minor chromosome arms, and the number of major and minor alleles for the set of 295 genes that overlap between COSMIC (12) and MSigDB (13). For both gene and arm-level events, we create a separate indicator for loss of heterozygosity on each gene.

Driver Mutations From CHASMplus (10) we gather the mutations on all COSMIC (12) oncogenes/tumor suppresor genes and binarize the presence or absence of a mutation in each gene.

C. Baselines. We are not aware of any other scalable meta-learning, deep learning, or varying-coefficient methods to produce context-informed correlation, Markov, and neighborhood selection networks under a universal framework. State-of-the-art gene regulatory network estimators are limited to population, cohort, and cluster-based approaches tailored to a single network model class (14–16). As such, our baselines apply the network estimators in 2 under several well-known and general paradigms for improving model personalization, broadly relating to subpopulation or cluster analysis. Our population baseline provides no personalization, learning a single model for the entire population of training samples. Our context-clustered baseline takes an unsupervised approach to personalization by first doing a k-means clustering with k=25 on the aggregated context views (3) and then inferring cluster-specific networks. Our disease-clustered baseline uses a personalization oracle, grouping samples by tumor type and then inferring disease-specific networks.

4. Extra Results

[*] www.cancer.gov/tcga

A. Simulations. We perform simulations to study the scaling properties of contextualized networks over network size (Fig. S1a, S2a) and context dimensionality (Fig. S1b, S2b).

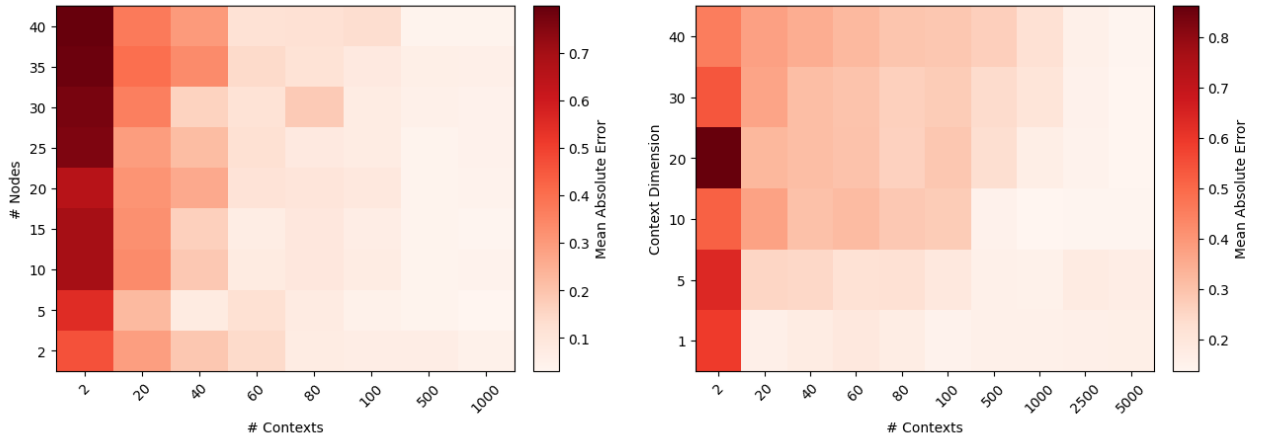

(a) Contextualized correlation network performance scaling by network size and total contexts

(b) Contextualized correlation network performance scaling by context dimensionality and total contexts

Fig. S1. Performance of estimators on held-out data, measured across (a) network size and (b) context dimensionality (i.e. complexity of context dependence) measured as the Mean-absolute Error (MAE) between predicted and ground truth correlation networks, averaged over three bootstrapped runs.

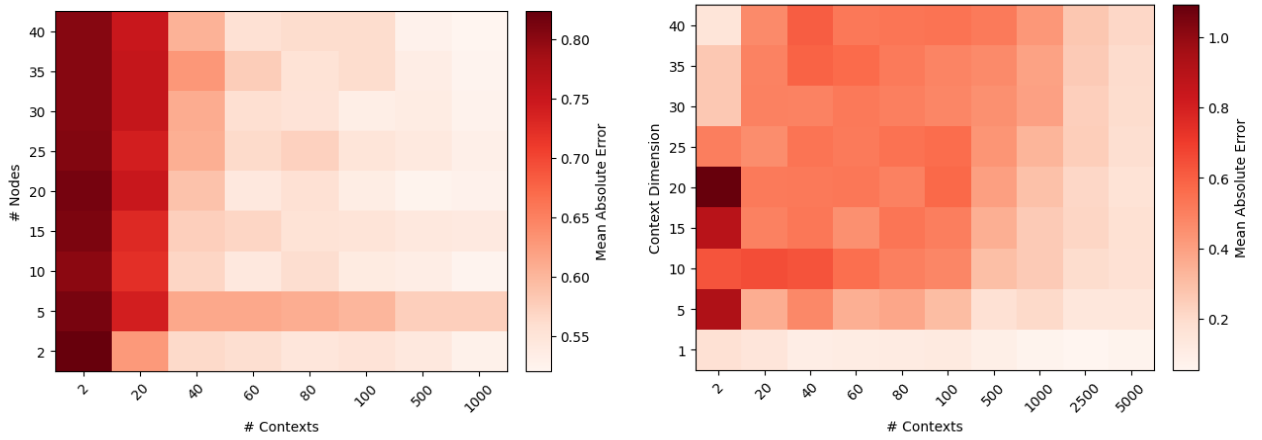

(a) Contextualized Markov network performance scaling by network size and total contexts

(b) Contextualized Markov network performance scaling by context dimensionality and total contexts

Fig. S2. Performance of estimators on held-out data, measured across (a) network size and (b) context dimensionality (i.e. complexity of context dependence) measured as the Mean-absolute Error (MAE) between predicted and ground truth Markov networks, averaged over three bootstrapped runs.

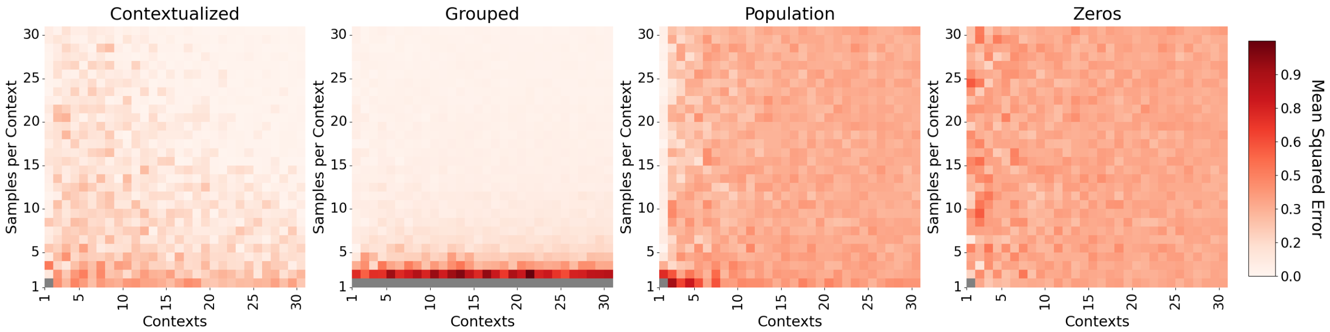

Fig. S3. Performance of correlation network estimators in terms of predicted versus true network parameters, measured over samples per context task and total contexts. Mean-squared error (MSE) is between the predicted and ground truth correlation matrices defining the correlation networks, averaged over five bootstrapped runs. Population estimates a single model for all contexts, grouped estimates a model for each context separately. Contextualization is the only method to scale both horizontally and vertically.

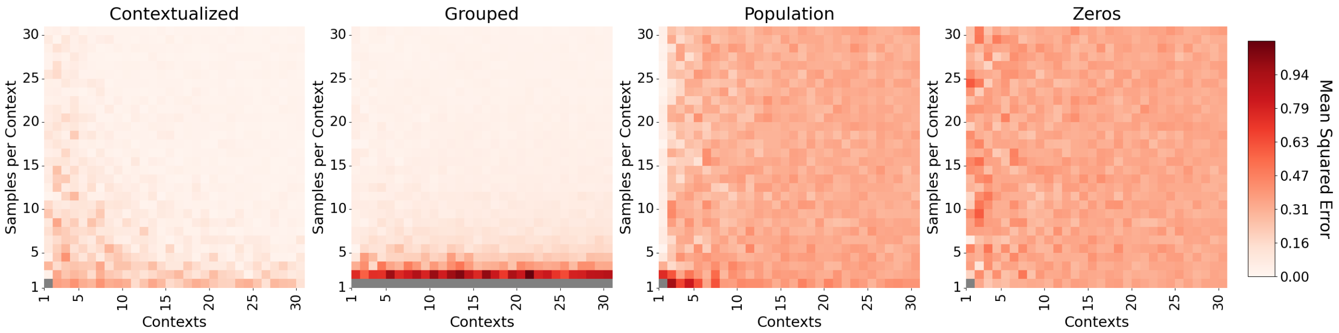

Fig. S4. Performance of Markov network estimators in terms of predicted versus true network parameters, measured over samples per context task and total contexts. Mean-squared error (MSE) is between the predicted and ground truth precision matrices defining the Markov networks, averaged over five bootstrapped runs. Population estimates a single model for all contexts, grouped estimates a model for each context separately. Contextualization is the only method to scale both horizontally and vertically.

Disease Type	Reference Subtypes	Expression Subtypes	Network Subtypes
Bladder Urothelial Carcinoma	0.411	0.151	0.713
Breast invasive carcinoma	1.484	0.616	1.558
Cholangiocarcinoma	–	–	–
Colon adenocarcinoma	0.016	0.014	1.219
Lymphoid Neoplasm Diffuse Large B-cell Lymphoma	–	–	–
Esophageal carcinoma	0.044	0.884	0.049
Glioblastoma multiforme	1.644	0.958	0.101
Head and neck squamous cell carcinoma	1.209	0.312	3.465
Kidney Chromophobe	0.715	13.802	0.211
Kidney renal clear cell carcinoma	5.042	6.109	13.741
Kidney renal papillary cell carcinoma	14.538	10.582	15.205
Brain Lower Grade Glioma	48.338	33.438	49.681
Liver hepatocellular carcinoma	0.009	0.427	0.827
Lung adenocarcinoma	0.687	1.172	0.507
Lung squamous cell carcinoma	0.123	0.105	0.249
Ovarian serous cystadenocarcinoma	0.704	0.684	0.05
Pancreatic adenocarcinoma	0.439	1.104	1.494
Prostate adenocarcinoma	–	–	–
Rectum adenocarcinoma	0.221	0.203	0.117
Skin cutaneous melanoma	–	–	–
Stomach adenocarcinoma	0.044	1.117	0.575
Thyroid adenocarcinoma	0.298	0.164	2.104
Uterine Corpus Endometrial Carcinoma	6.937	3.343	7.07
Uterine Carcinosarcoma	0.319	0.023	0.048
Uveal Melanoma	4.838	2.589	3.565

Table S1. Multivariate log-rank test comparison across different subtyping methods in terms of -log(p-value). Only samples shared between all datasets are used to control for power. – indicates no samples are shared, or subtypes do not exist for TCGA.

Disease Type	Reference Subtypes	Expression Subtypes	Network Subtypes
Bladder Urothelial Carcinoma	1.059	0.589	0.949
Breast invasive carcinoma	2.056	1.13	2.542
Cholangiocarcinoma	–	–	–
Colon adenocarcinoma	0.162	0.159	2.026
Lymphoid Neoplasm Diffuse Large B-cell Lymphoma	–	–	–
Esophageal carcinoma	0.323	1.598	0.284
Glioblastoma multiforme	1.631	1.509	0.63
Head and Neck squamous cell carcinoma	1.855	0.853	3.307
Kidney Chromophobe	0.715	13.802	0.211
Kidney renal clear cell carcinoma	5.61	5.247	14.82
Kidney renal papillary cell carcinoma	19.696	9.241	18.661
Brain Lower Grade Glioma	36.533	25.894	40.656
Liver hepatocellular carcinoma	0.105	0.619	1.263
Lung adenocarcinoma	1.67	2.29	1.198
Lung squamous cell carcinoma	0.614	0.417	0.625
Ovarian serous cystadenocarcinoma	1.414	1.325	0.287
Pancreatic adenocarcinoma	0.937	1.465	2.18
Prostate adenocarcinoma	–	–	–
Rectum adenocarcinoma	0.431	0.723	0.474
Skin Cutaneous Melanoma	–	–	–
Stomach adenocarcinoma	0.469	1.777	1.428
Thyroid carcinoma	0.837	0.831	3.242
Uterine Corpus Endometrial Carcinoma	5.555	3.319	7.42
Uterine Carcinosarcoma	0.319	0.023	0.048
Uveal Melanoma	5.076	2.61	4.536

Table S2. Minimum pairwise log-rank test comparison across different subtyping methods in terms of -log(p-value). Only samples shared between all datasets are used to control for power. – indicates no samples are shared, or subtypes do not exist for TCGA.

	Expression	Reference	Network
Bladder	0.680	0.483	0.413
Brain	0.857	0.876	0.871
Breast	0.718	0.597	0.773
Colorectal	0.364	0.429	0.551
Esophagus	0.398	0.532	0.496
Eye	0.500	0.618	0.867
Head and Neck	0.558	0.587	0.572
Kidney	0.717	0.609	0.697
Liver	0.646	0.452	0.588
Lung	0.432	0.558	0.497
Ovary	0.412	0.591	0.536
Pancreas	0.468	0.634	0.542
Thyroid	0.475	0.413	0.580
Skin	0.424	0.436	0.333
Stomach	0.620	0.545	0.520
Uterus	0.767	0.742	0.632

Table S3. Concordance index of survival regressors for each tissue trained on different patient representations.

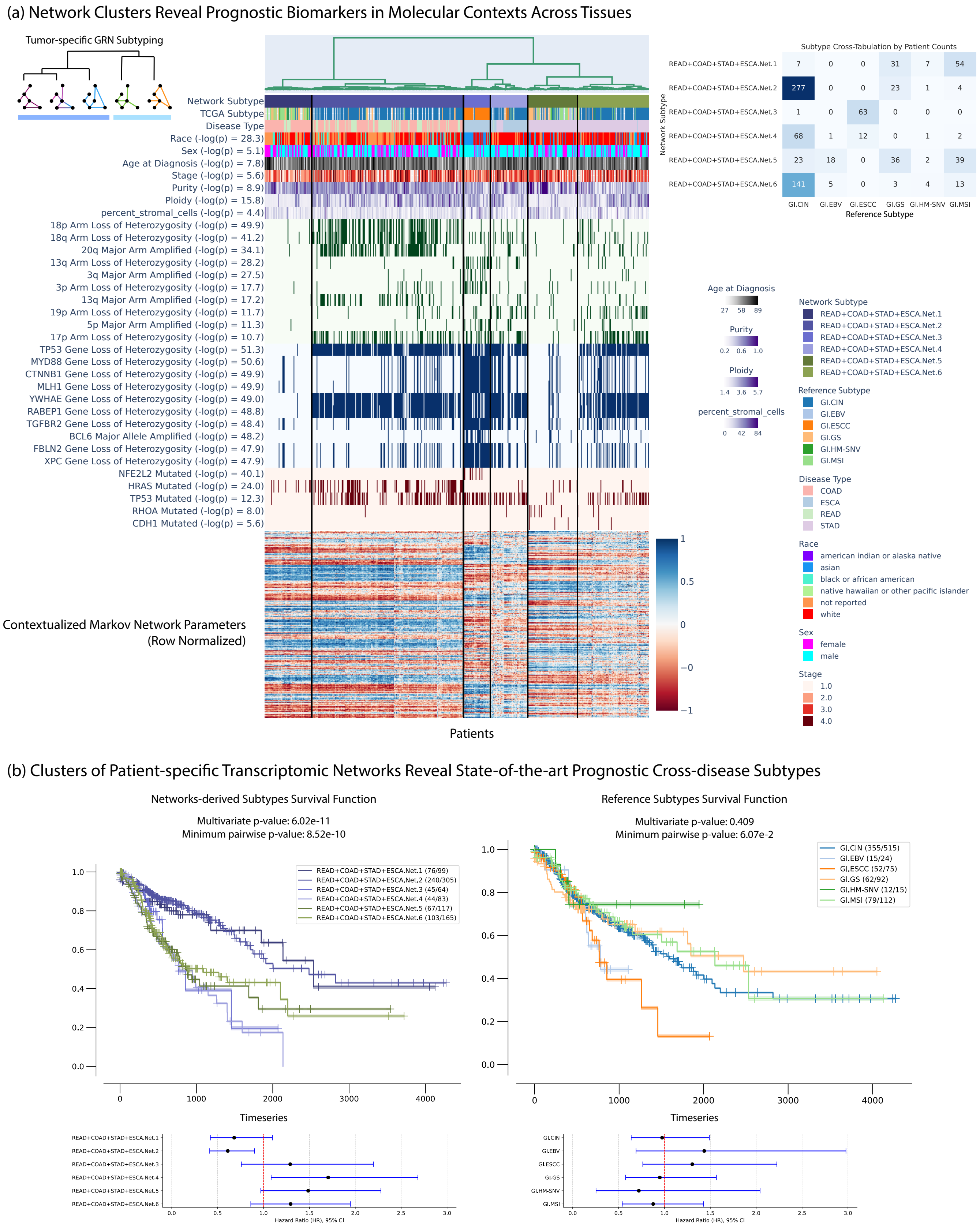

Fig. S5. Exploration of cross-disease network subtypes for cancers of the GI tract, including rectum adenocarcinoma, colon adenocarcinoma, stomach adenocarcinoma, and esophageal carcinoma, looking at correlated clinical information, arm-level copy alterations, gene-level copy alterations, and gene-level single nucleotide variations. Reference subtypes from (17).

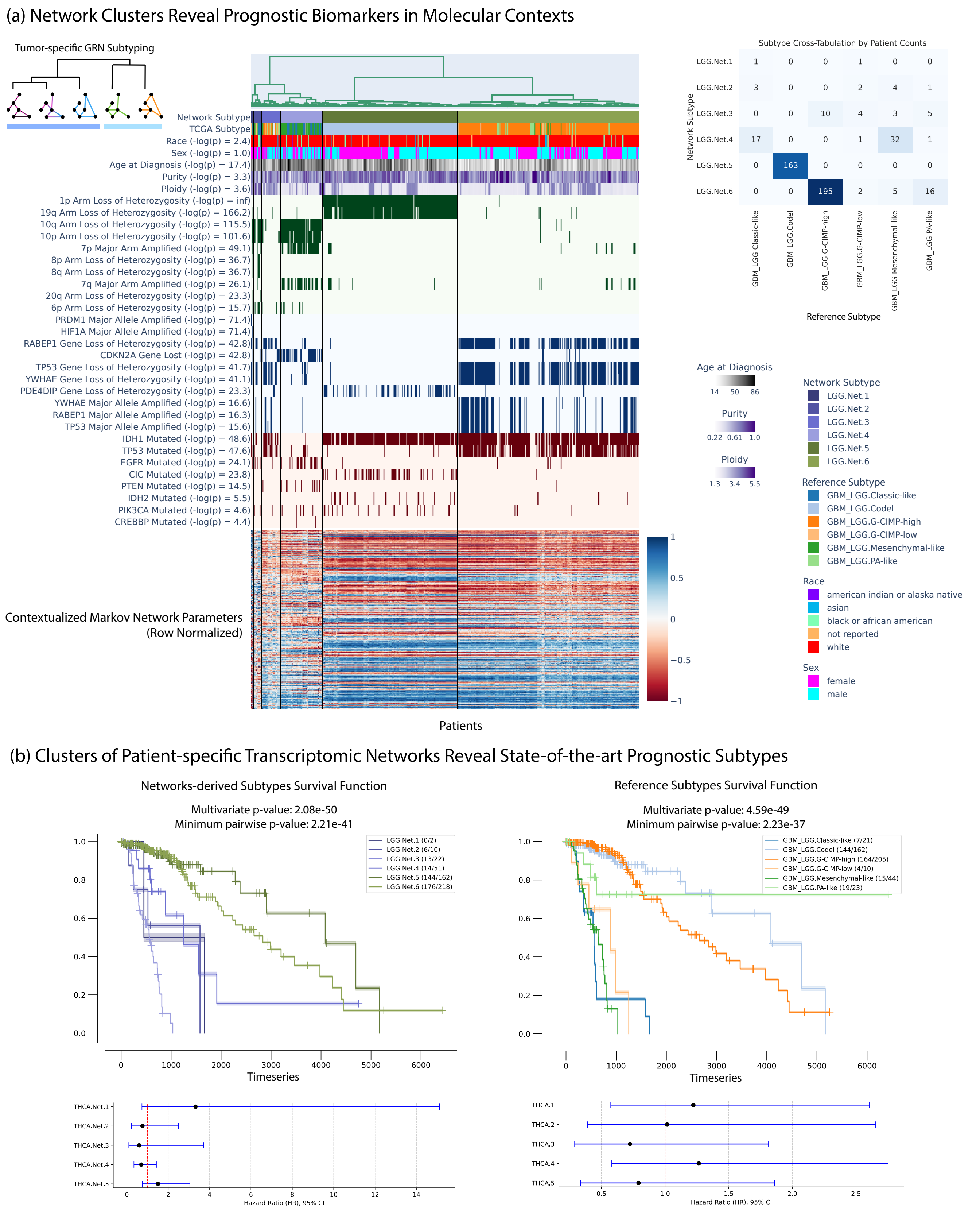

Fig. S6. Exploration of Brain Lower Grade Glioma, looking at correlated clinical information, arm-level copy alterations, gene-level copy alterations, and gene-level single nucleotide variations. Reference subtypes from (18).

SI Dataset S1 (data.tar.gz)

Pre-processed de-identified data from TCGA. https://zenodo.org/records/14885352/files/data.tar.gz

SI Dataset S2 (results.tar.gz)

Pre-trained contextualized networks (correlation, Markov, neighborhood) for all 7997 patients with train/test split labels. Includes subtyping plots for all 25 disease types with contextualized Markov networks. https://zenodo.org/records/14885352/files/results.tar.gz

References

1. CN Ellington, et al., Contextualized: Heterogeneous Modeling Toolbox. *J. Open Source Softw.* **9**, 6469 (2024).
2. R Agarwal, et al., Neural Additive Models: Interpretable Machine Learning with Neural Nets. (2021).
3. BJ Lengerich, ME Nunnally, Y Aphinyanaphongs, C Ellington, R Caruana, Automated Interpretable Discovery of Heterogeneous Treatment Effectiveness: A COVID-19 Case Study. *J. Biomed. Inform.* p. 104086 (2022).
4. BJ Lengerich, et al., Discriminative Subtyping of Lung Cancers from Histopathology Images via Contextual Deep Learning. *medRxiv* (2020) Publisher: Cold Spring Harbor Laboratory Press.
5. J Deuschel, et al., Contextualized Policy Recovery: Modeling and Interpreting Medical Decisions with Adaptive Imitation Learning (2023) arXiv:2310.07918 [cs, stat].
6. TI Zack, et al., Pan-cancer patterns of somatic copy number alteration. *Nat. Genet.* **45**, 1134–1140 (2013).
7. C Calabrese, et al., Genomic basis for RNA alterations in cancer. *Nature* **578**, 129–136 (2020) Number: 7793 Publisher: Nature Publishing Group.
8. P Van Loo, et al., Allele-specific copy number analysis of tumors. *Proc. Natl. Acad. Sci.* **107**, 16910–16915 (2010) Publisher: Proceedings of the National Academy of Sciences.
9. RM Drews, et al., A pan-cancer compendium of chromosomal instability. *Nature* **606**, 976–983 (2022) Number: 7916 Publisher: Nature Publishing Group.
10. C Tokheim, R Karchin, CHASMplus Reveals the Scope of Somatic Missense Mutations Driving Human Cancers. *Cell Syst.* **9**, 9–23.e8 (2019).
11. CD Steele, et al., Signatures of copy number alterations in human cancer. *Nature* **606**, 984–991 (2022) Number: 7916 Publisher: Nature Publishing Group.
12. Z Sondka, et al., The COSMIC Cancer Gene Census: describing genetic dysfunction across all human cancers. *Nat. Rev. Cancer* **18**, 696–705 (2018) Number: 11 Publisher: Nature Publishing Group.
13. A Liberzon, et al., The Molecular Signatures Database (MSigDB) hallmark gene set collection. *Cell Syst.* **1**, 417–425 (2015).
14. E Kim, V Gheorge, T Hart, Dynamic rewiring of biological activity across genotype and lineage revealed by context-dependent functional interactions. Publication Title: bioRxiv (2021).
15. S Mohammadi, J Davila-Velderrain, M Kellis, Reconstruction of Cell-type-Specific Interactomes at Single-Cell Resolution. *Cell Syst* **9**, 559–568.e4 (2019).
16. M Stone, et al., Identifying strengths and weaknesses of methods for computational network inference from single cell RNA-seq data. Publication Title: bioRxiv (2021).
17. Y Liu, et al., Comparative Molecular Analysis of Gastrointestinal Adenocarcinomas. *Cancer cell* **33**, 721–735.e8 (2018).
18. M Ceccarelli, et al., Molecular Profiling Reveals Biologically Discrete Subsets and Pathways of Progression in Diffuse Glioma. *Cell* **164**, 550–563 (2016).
